# Interleukin-18 in lupus nephritis: a meta-analysis of cytokine signaling dysregulation in immune-mediated nephropathy

**DOI:** 10.3389/fimmu.2025.1631728

**Published:** 2025-10-10

**Authors:** Cheng Zhou, Jiayi Li, Jing Zhao, Xue-Yuan Bai, Shunlai Shang, Wenge Li

**Affiliations:** ^1^ Department of Nephrology, China-Japan Friendship Hospital, Beijing, China; ^2^ Department of Nephrology, First Medical Center, Chinese People's Liberation Army (PLA) General Hospital, State Key Laboratory of Kidney Diseases, National Clinical Medical Research Center of Chronic Kidney Diseases, Beijing, China

**Keywords:** lupus nephritis (LN), interleukin − 18 (IL-18), meta-analysis, immune-mediated nephropathy, network-meta-analysis

## Abstract

**Background:**

Lupus nephritis (LN), the most severe renal complication in systemic lupus erythematosus (SLE), features inflammatory cascades from dysregulated cytokine networks. Although IL-18 (a pyroptosis effector) critically contributes to kidney inflammation, its dynamic changes across LN renal pathological stages and clinical correlations require systematic investigation. This meta-analysis explores peripheral blood IL-18’s association with renal pathological damage in LN, providing molecular insights into inflammatory signaling network imbalances.

**Study design and methods:**

This meta-analysis was performed to quantitatively assess the relationship between circulating IL-18 levels and lupus nephritis (LN) in SLE patients, incorporating stratified analyses by renal histopathological classifications (WHO classes II, III, IV, and V) to evaluate disease progression markers. We systematically queried multiple biomedical databases including PubMed, Embase, Scopus, Web of Science Core Collection, Wiley Online Library, MEDLINE, and Cochrane Library, encompassing all articles published before July 10, 2025. We pooled computed standardized mean difference (SMD) and its 95% confidence interval for meta-analytical using STATA 18.0.

**Results:**

A total of 18 eligible studies were included, 1,033 SLE with LN, 537 SLE without LN, and 1,083 matched healthy controls. The analysis results displayed that LN patients showed a significantly higher level of circulating IL-18 level in comparison with healthy controls (SMD = 2.51, 95% CI [1.91-3.12];I2 = 96.2%, p=0.000). The conclusion was equally applicable in subgroups divided based on sample type, mean age, disease duration, and testing method. SLE with LN patients showed a significantly higher level of circulating IL-18 level than SLE without LN(SMD = 1.53 95% CI [0.87–2.20]; I² = 95.7%, p =0.000). Compared with other classifications of LN, IV-LN patients demonstrate the highest serum IL-18 levels.

**Conclusions:**

This study reveals elevated IL-18 in LN patients, establishing it as a risk biomarker for SLE renal injury. The findings provide molecular evidence for IL-18 signaling imbalance in kidney inflammation and highlight therapeutic potential in targeting IL-18 to combat LN progression.

## Introduction

Lupus nephritis (LN), one of the most severe complications of systemic lupus erythematosus (SLE), affects 35%-60% of patients depending on factors such as race, gender, and age of onset and serves as a leading cause of morbidity and mortality (1). However, current measures for inducing remission or predicting flares in LN remain insufficiently effective, with up to 20% of LN patients ultimately progressing to end-stage renal disease within the first decade of their clinical course (2, 3).When patients develop clinical status changes (such as hematuria, proteinuria, or reduced renal function), a renal biopsy is required to confirm diagnosis. However, this procedure is not indicated for all patients (4).To address the therapeutic needs of LN, it is essential to identify biomarkers capable of accurately assessing disease risk and treatment response, including circulating inflammatory cytokines, immunocyte variations, and specific renal tissue proteins (5).

Abnormal cell death not only contributes to the pathogenesis of LN but also serves as a biomarker for predicting disease progression (6). Pyroptosis is a caspase-1-dependent proinflammatory programmed cell death characterized by cell membrane rupture and massive inflammatory factor release, closely linked to hyperinflammatory responses in LN kidney (7). Many studies have reported that enhanced pyroptosis in human and murine immune cells of SLE patients may further drive the development of nephritis and other lupus manifestations (8). Upon activation, the NLRP3 inflammasome releases IL-18 through caspase-1-mediated pyroptosis, exacerbating renal inflammation and fibrosis (9). IL-18, a hallmark inflammatory factor of pyroptosis, has been extensively implicated in playing a pivotal role in both SLE animal models and human disease (10, 11).

In the MRL/lpr lupus-prone mouse model, elevated IL-18 expression is detectable in the kidneys (12). Intraperitoneal administration of recombinant IL-18 has been shown to exacerbate nephropathy, whereas anti-IL-18 antibodies and IL-18 cDNA vaccines suppress IL-18 *in vivo*, thereby attenuating lymphocyte proliferation and nephritis (12). In renal biopsies from SLE patients, increased glomerular expression of IL-18 has been observed, and local production of this cytokine has been reported to play a significant role in driving dendritic cell migration to the kidneys (13). Elevated levels of IL-18 and IL-18BP (IL-18 binding protein) have been detected in patient serum, particularly in patients with active disease (14). Certain polymorphisms in the IL-18 gene are also associated with the development of SLE and LN (15). Therefore, we hypothesize that IL-18 and IL-18 derivatives (IL-18BP and free IL-18) may serve as a biomarker for SLE with LN.

Although several studies and meta-analyses have elucidated the role of IL-18 in SLE, a robust meta-analysis to investigate the correlation between circulating IL-18 and LN onset/staging is still needed. Here, we performed this meta-analysis aimed at pooling reported data and clarifying IL-18’s correlation with LN patients, providing evidence for its evaluation in disease risk and therapeutic response assessment.

## Methods

### Searching strategies

We searched medical databases including PubMed, Embase, Scopus, Web of Science Core Collection, Wiley Online Library, MEDLINE, and Cochrane Library, encompassing all articles published before July 10, 2025. Searching terms were a combination of “IL-18”,”IL-18BP”, “plasma”, “serum”, “Lupus nephritis”, “human”, and “patient”, and their corresponding free words were applied. Strategies were adapted according to different searching requirements for previously mentioned databases. Detailed searching queries are provided in the **Supplementary Table S1**. All the literature retrieval procedures were carried out by two authors (Cheng Zhou and Jiayi Li) independently. If the full text cannot be retrieved directly, we would contact study authors to get more information. All the procedures followed Preferred Reporting Items for Systematic Reviews and MetaAnalyses (PRISMA). We defined PICOS criteria in Meta-Analysis: Population-SLE patients with biopsy-proven LN (WHO/ISN-RPS Class II–V); Intervention/Exposure-Circulating IL-18 levels (serum/plasma); Comparator-(a) Healthy controls, (b) SLE without LN, (c) LN subclasses; Outcomes-Standardized mean difference (SMD) of IL-18 levels, correlation with histopathological class; study design–case–control, cohort, and cross-sectional studies.

### Inclusion and exclusion criteria

Articles which met the following standards were going to be contained for further data processing: (1) patients diagnosed as LN under a definite standard and matched healthy controls had been recruited; (2) circulating IL-18 level from LN patients and healthy controls were measured and reported either in plasma or serum; (3) study types including the cohort, case–control, and cross section; (4) the content was written in English. There were not any restrictions on ethnicity.

We had removed reviews, conference abstracts, editorials, commentaries, case reports, and studies performed on non-humans for lack of required information. Studies had also been exempted if LN patients were pregnant, infected or with other autoimmune diseases. As for repeated or duplicated studies, the more comprehensive and updated one would be selected.

### Inclusion and exclusion criteria

Articles which met following standards were going to be contained for further data processing: (1) patients diagnosed as LN under a definite standard and matched healthy controls had been recruited; (2) circulating IL-18 level from LN patients and healthy controls were measured and reported either in plasma or serum; (3) study types including the cohort, case control, and cross section; (4) the content was written in English. There were not any restrictions on ethnicity. We had removed reviews, conference abstracts, editorials, commentaries, case reports, and studies performed on non-humans for lack of required information. Studies had also been exempted if LN patients were pregnant, infected, or with other autoimmune diseases. As for repeated or duplicated studies, the more comprehensive and updated one would be selected.

### Data extraction and quality assessment

We reviewed all potentially eligible articles again in order to extract useful data and information, including author, region, year of publication, ethnicity, study design, number, mean age, gender, sample type, IL-18 circulating level of LN patients and normal controls, measurement method, diagnostic standard of LN, and disease duration. Data were extracted directly if the study reported the mean and standard deviation of circulating IL-18 level. If values were presented in median, standard error, range, and interquartile range, they were converted into mean and standard deviation for analysis using previously reported formulae (16). In the meantime, for the sake of evaluating the study quality of each article, Newcastle–Ottawa Scale (NOS) criteria were exploited for case–control studies and cohort studies (17). The NOS criteria are composed of three assessing aspects, namely, selection, comparability, and outcome assessment with a highest score of 9. Agency for Health-care Research and Quality (AHRQ) cross-sectional study quality assessment which contained 11 elements was used for cross-sectional studies. If the aggregated NOS score or AHRQ assessment score is above 6, the study quality can be regarded as relatively reliable. Detailed scores are provided in the **Supplementary Tables S2**, **S3**. Two reviewers (JZ and SLS) completed the extraction independently, and the final consent was met in case of any disagreements with the intervention of another reviewer (JZ).

### Statistical analysis

We obtained the mean and standard deviation of circulating IL-18 level from the included studies and then calculated the standardized mean difference (SMD) and its 95% confidence interval. All SMD values were pooled and presented in the form of forest plot. As for research heterogeneity, the Cochran’s Q statistic and Higgins I-squared statistical analysis (I^2^=[(Q−df)/Q]×100%) was undertaken (18). If the p value is less than 0.05 or calculated I2 is over 50%, significant heterogeneity may exist and hereby a random-effect model would be used, or else the fixed-effect model ought to be utilized. Meta-regression and subgroup analysis were also performed to explore the source of heterogeneity. Publication bias was first evaluated by the symmetry of funnel plot, and then Egger’s linear regression test was tried to assess more precisely (19). If a significant publication bias was detected, the trim and fill method was conducted to yield an unbiased effect size through recomputing the probable missing studies. The sensitivity test was simultaneously made to ensure the stability of this meta-analysis. Furthermore, the network-meta-analysis was conducted to evaluate IL-18 levels across different LN pathological stages (20). This type of analysis, similar to network meta-analysis, has been used before in recent meta-analysis studies on tumor staging (21, 22). The entire data analysis process was completed using STATA 18.0 (STATA, College Station, TX). If the p value is under 0.05, it would be recognized as statistical significance. This systematic review was registered with PROSPERO (CRD420251022010).

## Results

### Basic features and quality assessment

**Figure 1:**
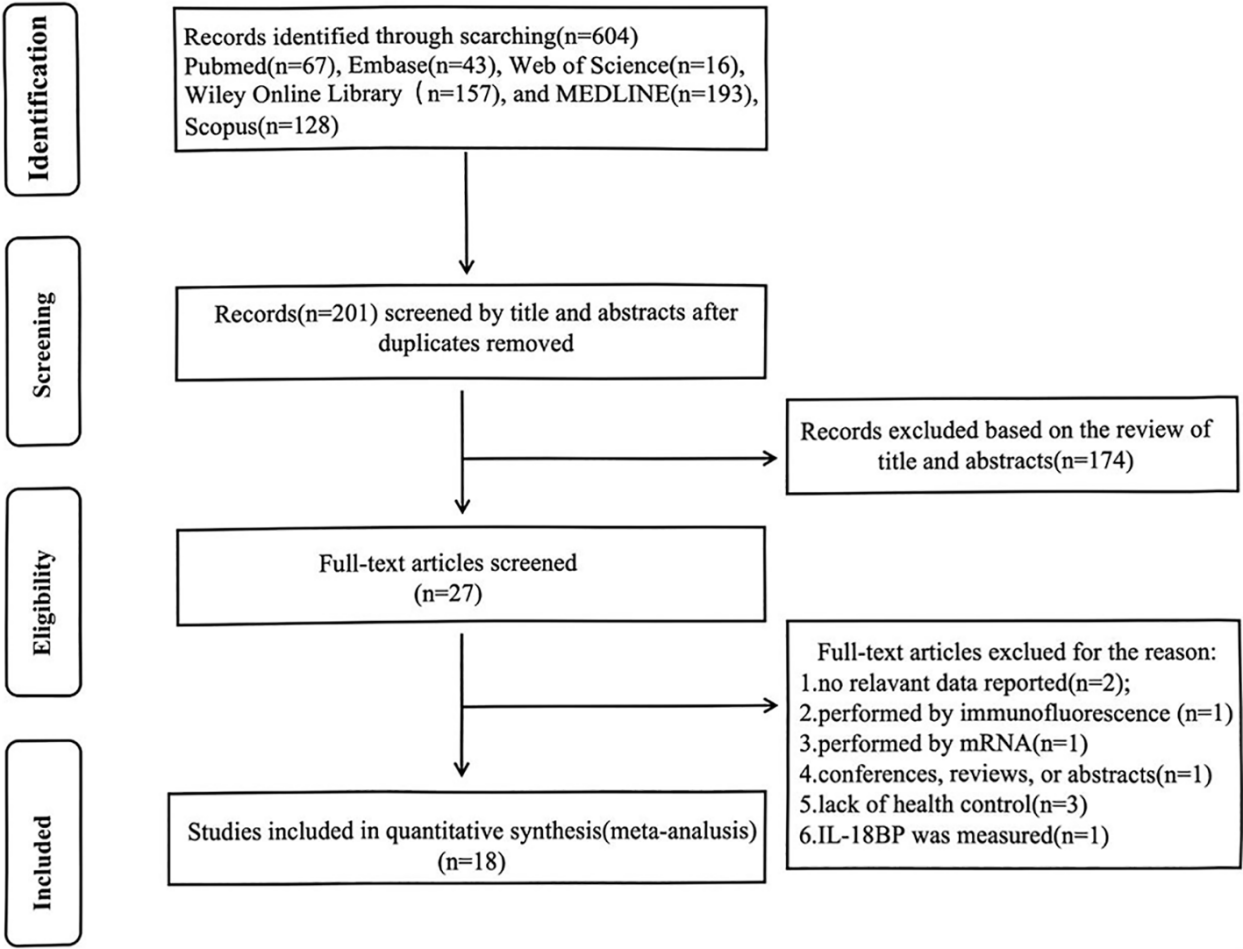
Flowchart of the inclusion and exclusion process.

Utilizing the search strategies listed above, a total of 604 studies were retrieved. First, duplicates were removed through title/abstract screening. After full-text review, studies that did not measure IL-18 levels or confirm LN diagnosis were excluded, ultimately identifying 18 articles meeting inclusion criteria. The flowchart of the detailed inclusion and exclusion process is displayed in **Figure 1**.With sample sizes varying from 26 to 402, a total of 1,033 SLE patients with LN, 537 without LN, and 1,083 normal controls were taken under analysis. These studies were conducted in 12 regions from 2002 to 2022, covering Asian, European, Middle Eastern, American, and North African populations. As for study quality assessment, most of the included studies can be recognized as reliable quality by NOS scale or AHRQ assessment. All relevant information is listed in **Table 1** (14, 15, 23–38). Of these, 11 studies have analyzed the relationship between IL-18 and LN staging (**Supplementary Table S4**). After excluding studies with incomplete IL-18 data and those containing single-case pathological staging reports, six studies were ultimately included in the network meta-analysis for LN staging (**Supplementary Table S5**).

**Table 1:** Basic characteristics of included 18 studies.

Author	Year	Region	Race	Study design	Study quality	Testing method	Sample type	Healthy controls				LN patients					
								Size	Mean age (y)	Sex (F/M)	IL-18 (pg/ml)	Size	Mean age (y)	Sex (F/M)	IL-18 (pg/ml)	Diagnose criteria	Disease duration (y)
Rachel Mende (23)	2018	Australian	Mixed	PC	8	ELISA	Serum	52	36	39/16	176.78 (±96.08)	58	44.9	NP	312.98 (±174.02)	ACR or SLICC	10.2
Xiaoqian Liu (24)	2010	China	Asian	RC	8	ELISA	Serum	20	34.3	17/3	48.00 (±45.00)	46	31.1	38/8	146.00 (±101.00)	ACR and WHO	NP
Marco Tucci (25)	2008	Italy	No Asian	CC	8	ELISA	Serum	41	NP	NP	150.00 (±25.00)	35	39.1	17/1	814.00 (±185.00)	WHO	NP
N.CALVANI (26)	2004	America Italy	Mixed	RC	8	ELISA	Serum	44	35.5	39/5	213.70 (±142.50)	61	36.9	53/8	736.90 (±447.80)	ACR and WHO	NP
K.R Sigdel (27)	2016	China	Asian	RC	8	Cytokine multiplex assay	Serum	24	33.37	21/3	11.86 (±3.27)	49	37.42	45/4	54.48 (±27.25)	ACR and WHO	NP
Dawei Hu (28)	2010	China	Asian	CC	8	ELISA	Serum	20	NP	NP	47.84 (±44.59)	46	31.7	38/8	146.42 (±100.98)	ACR and WHO	NP
Chie Shimizu (29)	2012	Japan	Asian	RC	8	ELISA	Serum	32	37	NP	244.00 (±24.00)	45	41	40/5	762.56 (±444.00)	ACR and ISN/RPS	5
C.K. WONG (30)	2002	Hong Kong	Asian	RC	8	ELISA	Plasma	28	38.5	27/1	135.49 (±87.96)	35	39.1	34/1	249.46 (±192.16)	ARA	12.4
Chao-Yi Wu (31)	2016	Taiwan	Asian	PC	9	ELISA	Serum	47	NP	NP	151.71 (±120.95)	65	12.56	58/7	849.20 (±110.71)	ACR and WHO/ ISN/RPS	10.42
N.A.K Ibrahim (32)	2020	Iraq	No Asian	RC	8	ELISA	Serum	40	41.02	NP	111.00 (±13.00)	40	31.05	32/8	896.00 (±134.00)	NP	6.11
V Umare (15)	2019	India	Asian	RC	7	ELISA	Serum	201	29.2	187/14	189.40 (±80.80)	109	28	NP	546.40 (±237.20)	ACR	2.3
R. J-N (33)	2016	Iran	No Asian	CC	8	ELISA	Serum	50	29.48	43/7	60.48 (±19.53)	99	30.74	92/7	649.58 (±663.72)	WHO	NP
A.R. EL. B (34)	2015	Egypt	No Asian	CC	8	ELISA	Serum	20	25.5	18/2	209.3 (±92.3)	40	26.3	35/5	612.42 (±169.60)	ISN/RPS	9.6
K.F Koenig (35)	2012	Switzerland	No Asian	PC	9	Cytokine multiplex assay	Serum	14	38	11/3	67.50 (±95.00)	12	31	8/4	332.83 (±357.66)	ACR	NP
Mona A. Mohsen (36)	2013	Egypt	No Asian	CC	9	ELISA	Serum	15	35.92	12/3	112.01 (±48.23)	41	32.41	32/9	156.13 (±57.86)	ACR	4.67
DY Chen (37)	2009	Taiwan	Asian	CC	8	ELISA	Serum	174	36.8	85/89	77.79 (±39.77)	101	31.8	92/9	347.19 (±222.70)	ACR	6.9
Alyaa Farid (38)	2022	Egypt	No Asian	CC	9	ELISA	Serum	250	43.5	218/32	76.80 (±23.90)	152	48.1	137/15	146.70 (±6.40)	SLICC	9
Dong Liang (14)	2006	China	Asian	CC	6	ELISA	Plasma	11	–	5/6	238.90 (±64.40)	16	–	15/1	767.00 (±133.50)	ACR	–

F/M, female versus male; PC, prospective cohort study; RC, retrospective cohort study; CC, cross-sectional cohort study; ELISA, enzyme linked immunosorbent assay; NP, not provided; ACR, American College of Rheumatology diagnostic standard; SLICC, Systemic Lupus International Collaborating Clinics; WHO, World Health Organization classification of lupus nephritis; ISN/RPS, International Society of Nephrology/Renal Pathology Society classification of lupus nephritis; ARA, American Rheumatism Association criteria.

### LN patients had high levels of circulating IL−18 compared with healthy controls

**Figure 2:**
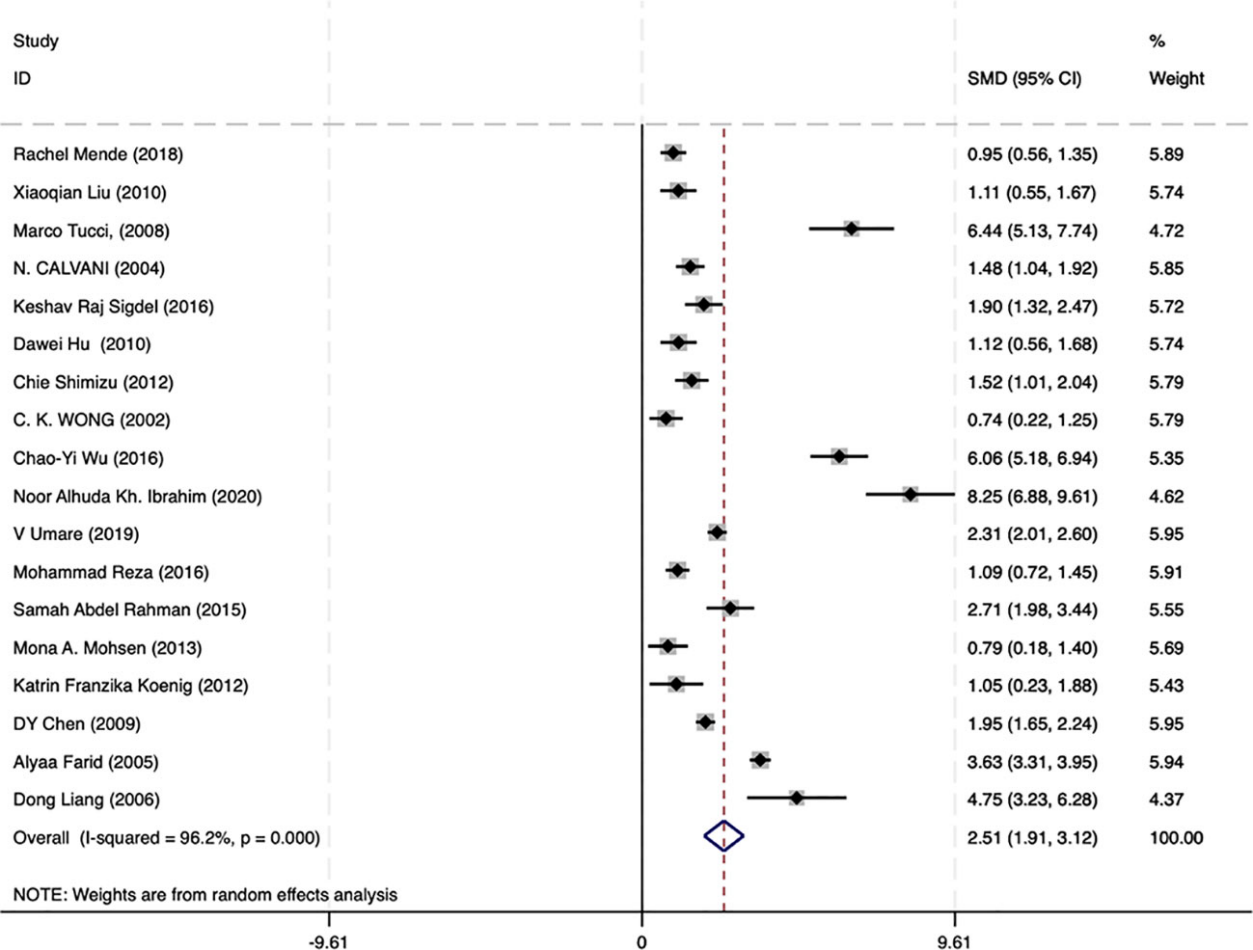
Forest plot of the standard mean variance (SMD) for the levels of circulating IL-18 in LN patients and healthy controls using a random-effect model.

**Figure 3:**
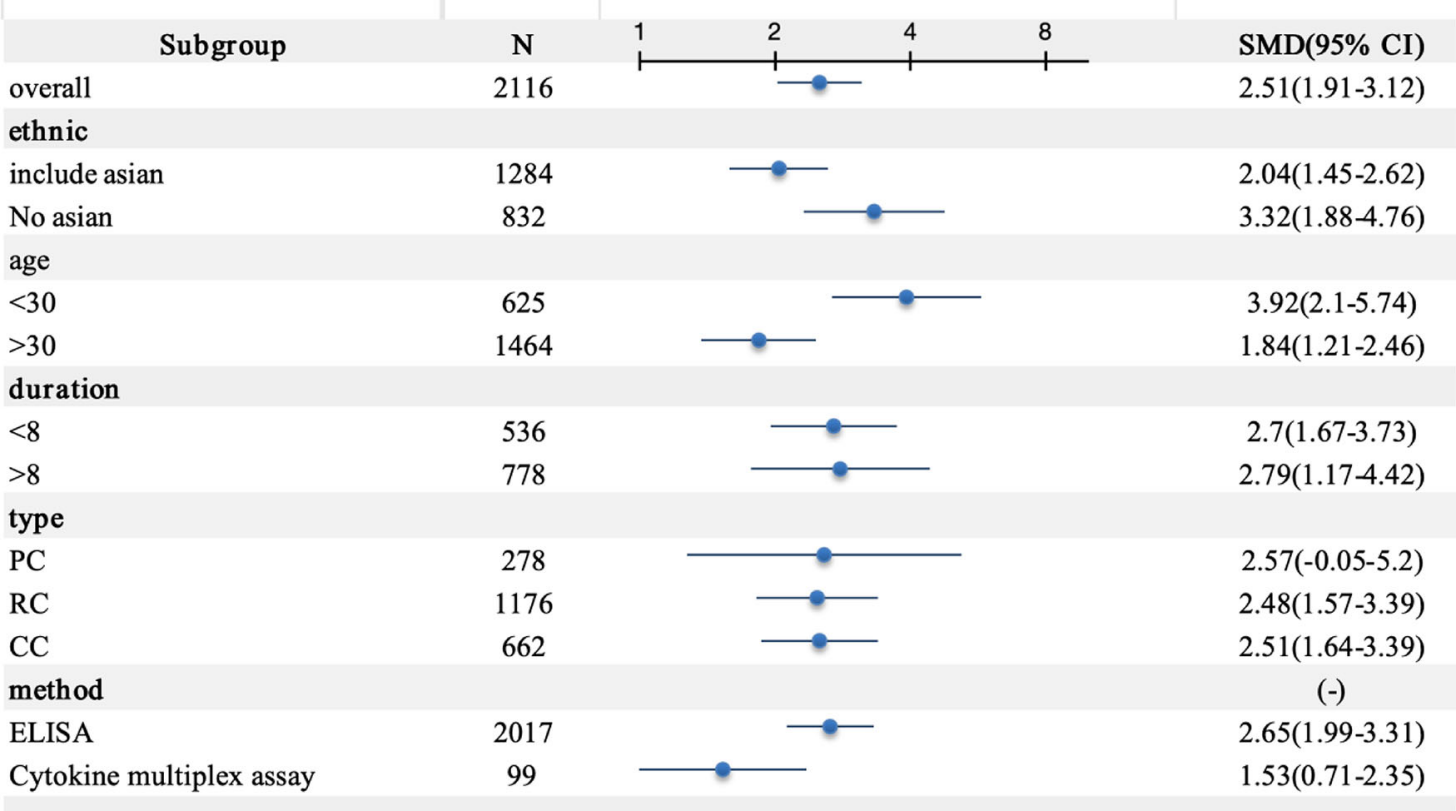
Subgroup meta-analysis of circulating interleukin-18 in LN patients. PC, prospective cohort study; RC, retrospective cohort study; CC, cross-sectional cohort study; ELISA, enzyme-linked immunosorbent assay.

A meta-analysis incorporating data from 18 studies revealed significantly elevated circulating IL-18 levels in LN patients compared with healthy controls (SMD = 2.51, 95% CI [1.93-3.12]). Given the substantial heterogeneity among studies (I^2^ = 96.2%, p=0.000), a random-effects model was implemented for the analysis (**Figure 2**). Subgroup analyses based on age, ethnicity, sample type, disease duration, and testing method meta-analysis results are summarized in **Figure 3**. The level of IL-18 was higher in the patients age ≤30 (SMD = 3.92, 95% CI [2.1-5.74] vs. SMD = 1.84, 95% CI [1.21-2.46]). Increased IL-18 had also been observed in all subgroups stratified by ethnicity (including the Asian ethnic population nor not), disease duration time (years ≤8 or >8), study type, and detection methods.

### The level of circulating IL−18 was closely related to the renal damage in SLE

**Figure 4:**
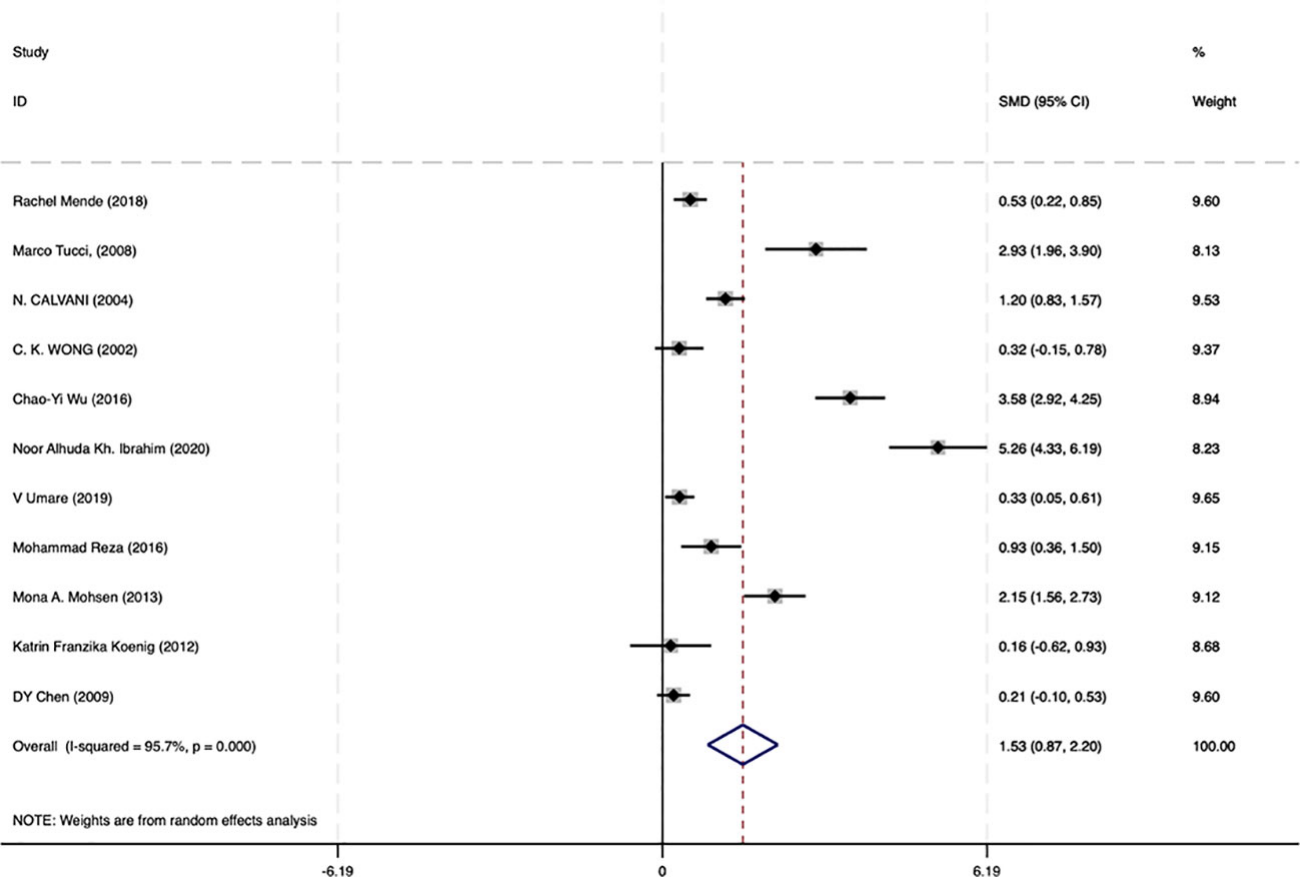
Forrest plot of the standard mean variance (SMD) for the levels of circulating IL-18 in SLE with LN patients and SLE without LN patients using a random-effect model.

Among the 17 studies, 11 analyzed circulating IL-18 levels in SLE with LN and SLE without LN. To further examine the correlation between IL-18 and renal lesions in SLE, we conducted statistical analysis on data from SLE with LN and SLE without LN patients in these 11 studies. The results demonstrated that circulating IL-18 levels were significantly higher in SLE with LN patients compared with SLE without LN patients (SMD = 1.53 95% CI [0.87–2.20]; I² = 95.7%, p =0.000) (**Figure 4**). These findings suggest that IL-18 levels are significantly elevated during renal injury in SLE.

### The level of circulating IL−18 was closely related to the renal damage in SLE

**Figure 5:**
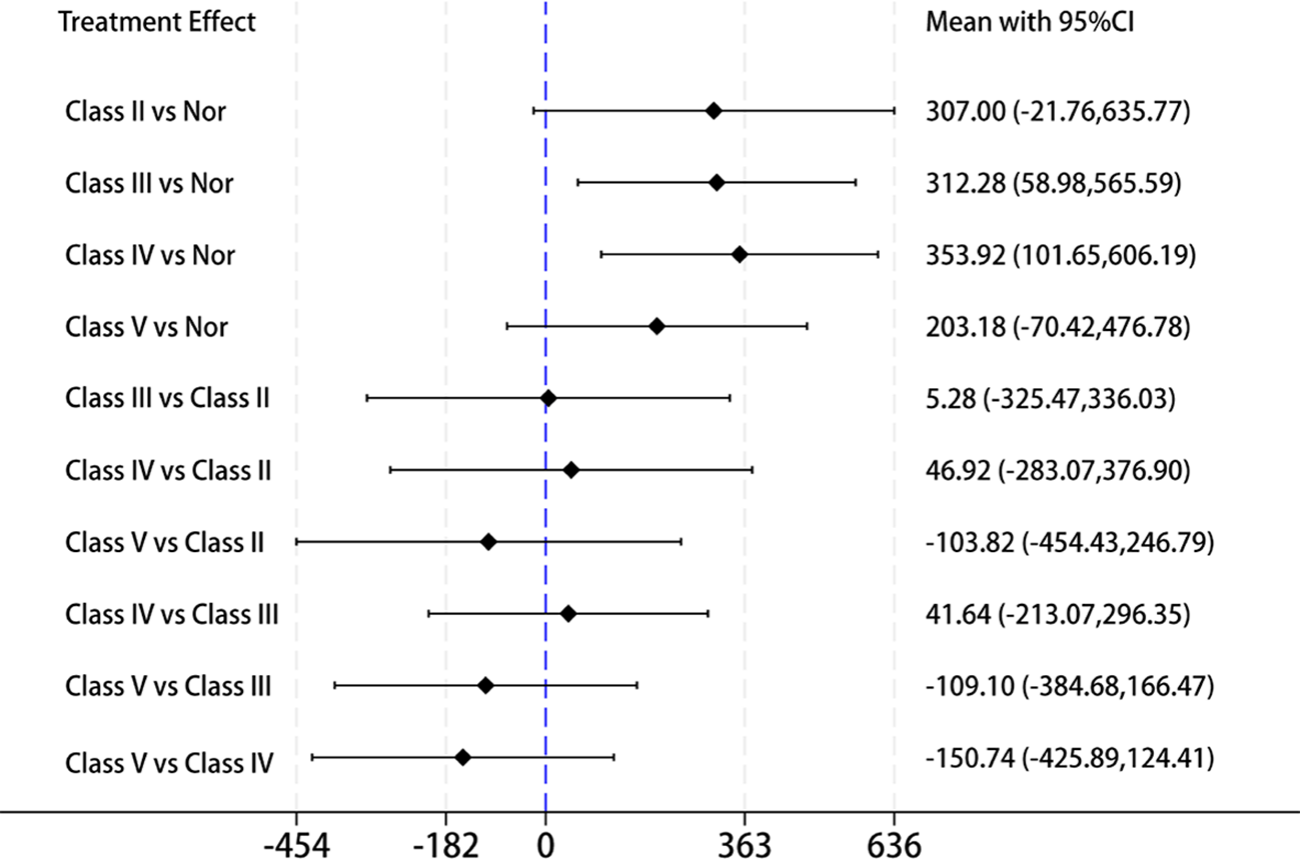
Forest plot of the mean with 95% CI for the levels of circulating IL-18 cross different LN pathological stage(classes II, III, IV, and V) using network meta-analysis.

Then, we extracted data from 6 of the 17 studies that included LN staging to further validate the correlation between IL-18 levels and the types of renal pathological damage in LN. Network meta-analysis is typically used to analyze pairwise comparisons between different treatment approaches or medications. In this study, we propose to employ a network meta-analysis strategy for pairwise comparisons to evaluate IL-18 levels across different LN pathological stages. Here, we used IL-18 levels in healthy individuals as a common control and compared them with LN classes II, III, IV, and V to determine the pathological stage with the highest IL-18 levels in LN. The results revealed that IL-18 levels were highest in Class IV-LN and lowest in Class V when compared with other LN classifications (**Figure 5**). Class IV-LN showed the most extensive glomerular involvement and corresponds to diffuse LN. These findings suggest that IL-18 levels are closely correlated with the degree of glomerular involvement in LN.

### Sensitivity analysis, publication bias, and meta−regression

**Figure 6:**
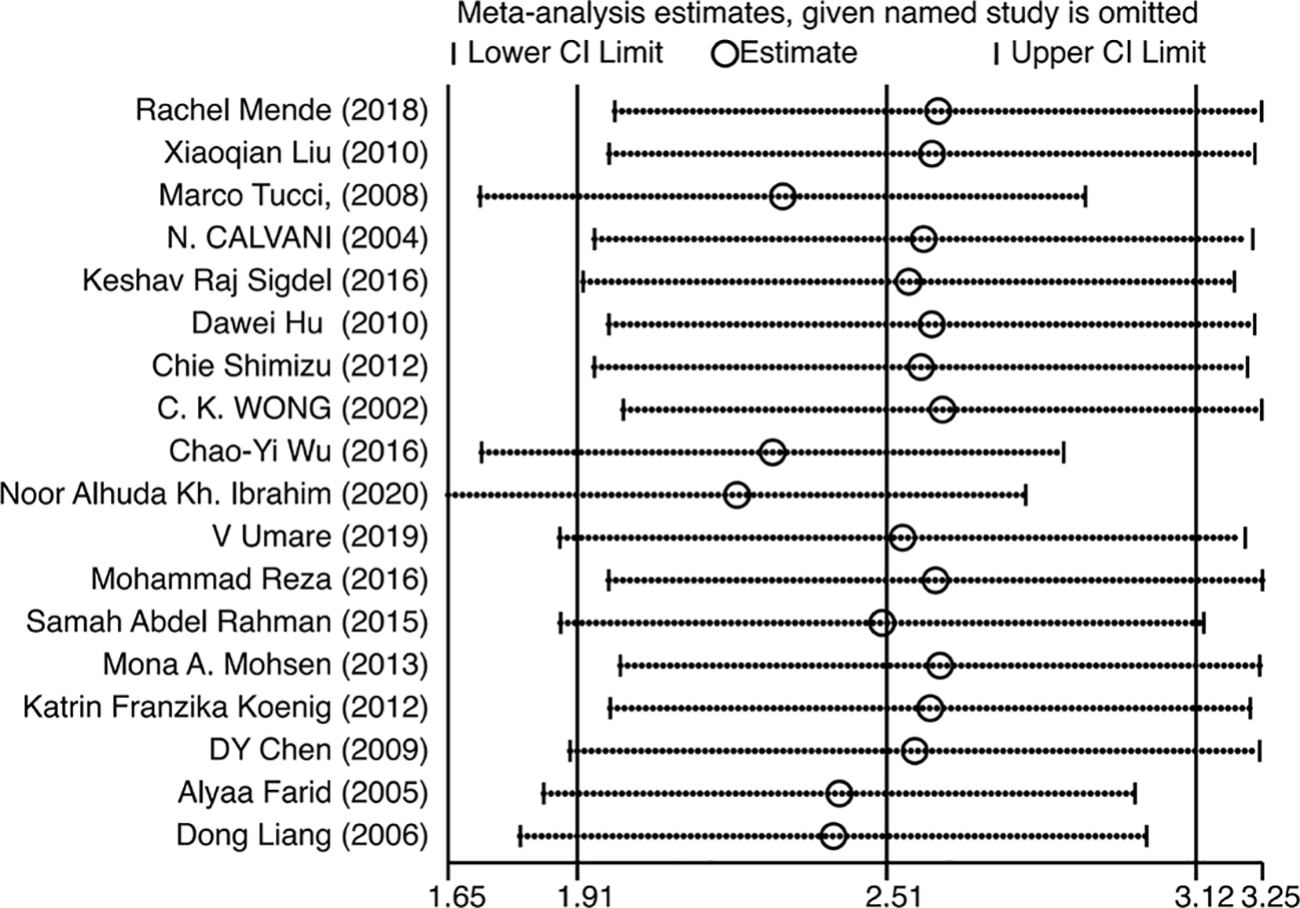
Sensitivity analysis of the pooled standard mean variance (SMD).

**Figure 7:**
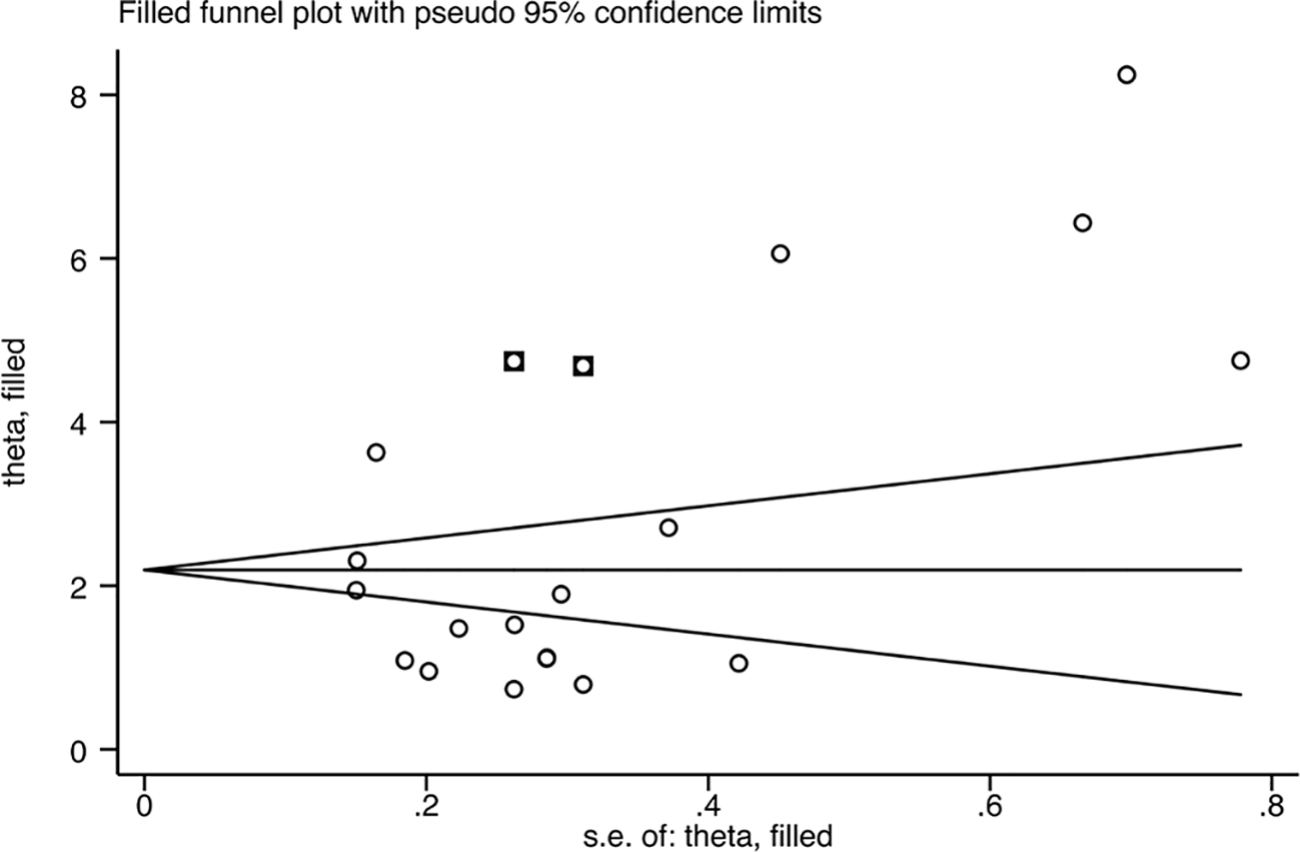
Funnel plot after filling in the potential missing studies using the trim and fill method.

Since the heterogeneity of the association between circulating IL-18 and LN was pronounced, an additional sensitivity test was done (**Figure 6**). There were no conspicuous alternations detected when removing one of the included studies and pooling the rest. No statistically remarkable bias was detected in circulating IL-18 levels between LN patients and healthy controls by Egger’s linear regression test (p>0.05) (**Supplementary Figure S6**). After filling in the two hypothetical missing studies, the adjusted SMD remained significant (SMD = 2.76, 95% CI [2.12–3.40], p<0.01), which was in accordance with previous outcomes (**Figure 7**). On the whole, it indicated that the primary results of this meta-analysis were relatively robust. To further explore the source of heterogeneity, meta-regression was utilized. With these variables, including publication year, mean age of LN groups, disease duration, sample type, study design, and ethnicity, contained in the regression model separately, the main meta results had not changed with p value >0.05 (**Supplementary Figure S7**).

## Discussion

This meta-analysis incorporated 18 published studies from 12 regions, encompassing 1,033 LN patients and 1,083 healthy controls. After processing all extracted relevant data, it was found that circulating IL-18 levels were significantly elevated in LN patients compared with healthy individuals, suggesting the potential role of IL-18 in the pathogenesis of LN. More importantly, when subgroup analyses were performed based on sample type, ethnicity, disease duration, patient age, and detection methods, this association remained statistically significant. The conclusions drawn from our meta-analysis are consistent with the majority of the included individual studies, which enhances the reliability. These findings suggest that IL-18 is closely associated with the onset of LN and may serve as a novel immunological biomarker for LN patients.

In lupus nephritis, dysregulated cytokines create a “cytokine storm” where IL-18—significantly elevated in serum (especially during active nephritis) and overexpressed in renal tubulointerstitium (correlating with inflammatory infiltration)—acts as a central hub by synergizing with IFN-γ, IL-1β, IL-12, IL-23, and BAFF to drive renal injury: promoting IFN-γ/Th1 responses with IL-12 (12), amplifying NLRP3 inflammasome cascades with IL-1β (39), enhancing Th17-mediated neutrophil recruitment (40), and forming a BAFF-driven autoreactive B-cell loop (41).

Several recent studies have reported correlations between IL-18 and disease severity, organ involvement, and a range of conventional biomarkers (e.g., anti-dsDNA antibodies, C3, and C4). IL-18 is also correlated with proteinuria and renal activity. Its elevation precedes the occurrence of proteinuria and high disease severity scores, indicating that elevated IL-18 levels are closely related to the development of renal damage in SLE. Data extraction and processing from 12 studies that measured circulating IL-18 levels in SLE patients without LN revealed that peripheral blood IL-18 levels were significantly higher in SLE with LN patients compared with those with SLE without LN.

IL-18, a pro-inflammatory cytokine in the IL-1 superfamily, critically contributes to SLE and lupus nephritis (LN). Meta-analysis demonstrates significantly elevated serum IL-18 levels in LN patients with active nephritis compared with renal-sparing SLE, highlighting its role as a key mediator of renal damage. Mechanistically, IL-18 drives renal inflammation by activating Th1/Th17 responses, enhancing IFN-γ production, and recruiting neutrophils into glomeruli. This inflammatory milieu correlates with IL-18’s pronounced expression in renal tissues, particularly glomerular endothelial cells and infiltrating macrophages, where it associates with severe proliferative lesions and adverse outcomes. Unlike traditional biomarkers such as anti-dsDNA antibodies—which primarily reflect systemic autoimmune status—IL-18 correlates more specifically and sensitively with renal inflammatory activity and pathological severity (e.g., in Class IV LN). Its serum levels often rise earlier than clinical proteinuria recurrence, providing a real-time predictive window for disease flares. The ISN/RPS classification further underscores IL-18’s stage-specific dynamics, reaching peak levels in Class IV LN (diffuse proliferative glomerulonephritis), which is characterized by endothelial injury and marked inflammatory cell infiltration. Supporting this, Class IV LN biopsy specimens show upregulated GSDMD-mediated pyroptosis and NLRP3 inflammasome activity, positioning IL-18 as both a biomarker and an effector of pyroptosis-driven renal injury. Recent advances elucidate the critical role of GSDMD-mediated pyroptosis in LN progression (8). NLRP3 inflammasome-activated caspase-1 cleaves GSDMD to form membrane pores, releasing IL-18 and IL-1β, which amplify inflammatory cascades. In Class IV LN, heightened macrophage pyroptosis correlates with elevated serum IL-18 levels, podocyte depletion, and histologic activity indices, suggesting IL-18’s dual role as a pyroptosis product and driver. Translating these insights, experimental inhibition of NF-κB or NLRP3 reduces pyroptosis and IL-18 release, thereby mitigating glomerular damage. Therapeutically, conventional therapies (e.g., corticosteroids and cyclophosphamide) and novel agents (mycophenolate mofetil combined with tacrolimus) can suppress IL-18 production or modulate the IL-18/IL-18BP balance. These findings collectively establish IL-18 as a pivotal player in LN pathogenesis, with its dual role in pyroptosis offering novel therapeutic avenues for intercepting disease progression.

IL-18 binding protein (IL-18BP) acts as a potent and specific antagonist of IL-18, neutralizing free IL-18 through high-affinity binding. Research by Novick et al. demonstrated that serum levels of IL-18BP and free IL-18 were significantly elevated in SLE patients compared with healthy controls, with increases of approximately six-, three-, and fivefold for IL-18, IL-18BP, and free IL-18, respectively (42). These levels correlated with disease activity as assessed by the SLEDAI-2K score. Furthermore, Favilli et al. revealed that serum-free IL-18 levels, rather than total IL-18, correlated with disease severity. In contrast, urinary analysis showed that only total IL-18 elevation was associated with disease severity, whereas free active IL-18 levels were not (43).

Among 18 studies investigating IL-18 in lupus nephritis (LN), only three concurrently measured serum IL-18BP levels. Liang et al. reported significantly elevated plasma IL-18 (767.0 ± 133.5 pg/ml vs. 238.9 ± 64.4 pg/ml, p < 0.01) and IL-18BP (8.0 ± 3.0 ng/ml vs. 5.6 ± 2.1 ng/ml, p < 0.05) levels in LN patients compared with healthy controls (14). Wu et al. found that serum IL-18 correlated significantly with serum IL-18BP and free IL-18 levels, but not with urinary IL-18. However, only serum IL-18 level, and not IL-18BP, free IL-18, or urinary IL-18, correlated with renal inflammatory activity in SLE patients (P = 0.033 vs. P = 0.192, P = 0.361, P = 0.605, respectively) (31). Shimizu et al. observed significantly higher serum IL-18BP levels in LN patients with more severe histological activity and chronicity (e.g., classes III and IV; P < 0.05), whereas levels in class II and V patients did not significantly differ (29).

Dysregulation of the IL-18/IL-18BP axis results in excessive bioactivity of free IL-18, a key mediator of inflammation in systemic lupus erythematosus (SLE) and lupus nephritis (LN). This meta-analysis quantitatively confirms elevated serum IL-18 levels, reflecting this underlying pathological imbalance. Therapeutically, neutralizing free IL-18 represents a promising strategy. Agents under clinical development include recombinant IL-18 binding protein (e.g., Tadekinig alfa) and neutralizing anti-IL-18 monoclonal antibodies, with early studies showing efficacy in autoimmune inflammatory conditions (44). Our finding of a strong association between elevated IL-18 and LN, particularly severe class IV disease, provides a rationale for exploring targeted IL-18 blockade in LN. Future studies incorporating concurrent measurement of free IL-18 and IL-18BP are essential to refining risk stratification based on this pathway and to monitoring responses to IL-18-targeted therapies (45, 46).

This meta-analysis has several limitations that warrant careful consideration. First, the inclusion of studies with relatively small sample sizes or those conducted in local clinical settings may compromise the quality and generalizability of the findings. Second, the credibility and data quality of the 18 included studies varied, as not all were published in high-impact journals. More importantly, the overwhelming reliance on cross-sectional designs significantly restricts the ability to infer causal relationships between IL-18 and lupus nephritis (LN), or to capture dynamic changes in IL-18 levels relative to disease activity, treatment response, and pathological outcomes. This design limitation also precludes mechanistic insight into how IL-18 may regulate intrarenal inflammation and downstream signaling pathways. Despite these limitations, the use of random-effects models and thorough subgroup and sensitivity analyses helped maintain the robustness of the main conclusions. Although this study has limitations such as relatively small sample sizes and a lack of longitudinal data, it precisely underscores the deficiencies in current cross-sectional research and provides a foundation for future large-scale multicenter, longitudinal studies aimed at clarifying the evolving role of IL-18 in the disease process.

## Conclusion

Elevated serum interleukin-18 (IL-18) in lupus nephritis (LN) correlates with renal histopathological severity, highlighting its potential as a prognostic biomarker and pathogenic driver. Elucidating IL-18’s signaling dynamics enhances understanding of cytokine interplay in autoimmune renal injury and aids in developing targeted therapies for LN. Future research must clarify IL-18’s role in renal cytokine networks to develop therapies targeting renal inflammation in systemic lupus erythematosus (SLE).

## Data Availability

The original contributions presented in the study are included in the article/**Supplementary Material**. Further inquiries can be directed to the corresponding author.

## References

[1] Anders HJ Saxena R Zhao MH Parodis I Salmon JE Mohan C . Lupus nephritis. Nat Rev Dis Primers. (2020) 6:7. doi: 10.1038/s41572-019-0141-9, PMID: 31974366

[2] Gou W Tuo YH . Comparison of mortality and its causes in patients with complicated systemic lupus erythematosus on hemodialysis versus peritoneal dialysis: A meta-analysis. Med (Baltimore). (2022) 101:e30090. doi: 10.1097/MD.0000000000030090, PMID: 35960069 PMC9371503

[3] Parodis I Houssiau FA . From sequential to combination and personalised therapy in lupus nephritis: moving towards a paradigm shift? Ann Rheum Dis. (2022) 81:15–9. doi: 10.1136/annrheumdis-2021-221270, PMID: 34521616

[4] Giannico G Fogo AB . Lupus nephritis: is the kidney biopsy currently necessary in the management of lupus nephritis? Clin J Am Soc Nephrol. (2013) 8:138–45. doi: 10.2215/CJN.03400412, PMID: 22977215 PMC12895570

[5] De Vriese AS Sethi S Fervenza FC . Lupus nephritis: redefining the treatment goals. Kidney Int. (2025) 107:198–211. doi: 10.1016/j.kint.2024.10.018, PMID: 39521057

[6] Mistry P Kaplan MJ . Cell death in the pathogenesis of systemic lupus erythematosus and lupus nephritis. Clin Immunol. (2017) 185:59–73. doi: 10.1016/j.clim.2016.08.010, PMID: 27519955 PMC5299061

[7] Hu B Ma K Wang W Han Z Chi M Nasser MI . Research progress of pyroptosis in renal diseases. Curr Med Chem. (2024) 31:6656–71. doi: 10.2174/0109298673255656231003111621, PMID: 37861024

[8] Cao H Liang J Liu J He Y Ke Y Sun Y . Novel effects of combination therapy through inhibition of caspase-1/gasdermin D induced-pyroptosis in lupus nephritis. Front Immunol. (2021) 12:720877. doi: 10.3389/fimmu.2021.720877, PMID: 34867948 PMC8639704

[9] Wu D Ai L Sun Y Yang B Chen S Wang Q . Role of NLRP3 inflammasome in lupus nephritis and therapeutic targeting by phytochemicals. Front Pharmacol. (2021) 12:621300. doi: 10.3389/fphar.2021.621300, PMID: 34489689 PMC8417800

[10] Xue D Qian Y Tu X He M Xing F Ren Y . The effect of circulating cytokines on the risk of systemic lupus erythematosus: Mendelian randomization and observational study. Immunogenetics. (2024) 76:315–22. doi: 10.1007/s00251-024-01351-x, PMID: 39183206 PMC11496328

[11] Yang CA Chiang BL . Inflammasomes and human autoimmunity: A comprehensive review. J Autoimmun. (2015) 61:1–8. doi: 10.1016/j.jaut.2015.05.001, PMID: 26005048

[12] Bossù P Neumann D Del Giudice E Ciaramella A Gloaguen I Fantuzzi G . IL-18 cDNA vaccination protects mice from spontaneous lupus-like autoimmune disease. Proc Natl Acad Sci U S A. (2003) 100:14181–6. doi: 10.1073/pnas.2336094100, PMID: 14615579 PMC283566

[13] Tucci M Calvani N Richards HB Quatraro C Silvestris F . The interplay of chemokines and dendritic cells in the pathogenesis of lupus nephritis. Ann N Y Acad Sci. (2005) 1051:421–32. doi: 10.1196/annals.1361.084, PMID: 16126984

[14] Liang D Ma W Yao C Liu H Chen X . Imbalance of interleukin 18 and interleukin 18 binding protein in patients with lupus nephritis. Cell Mol Immunol. (2006) 3:303–6., PMID: 16978540

[15] Umare V Pradhan V Nath S Rajadhyaksha A Ghosh K Nadkarni AH . Impact of functional IL-18 polymorphisms on genetic predisposition and diverse clinical manifestations of the disease in Indian SLE patients. Lupus. (2019) 28:545–54. doi: 10.1177/0961203319834677, PMID: 30857465

[16] Wan X Wang W Liu J Tong T . Estimating the sample mean and standard deviation from the sample size, median, range and/or interquartile range. BMC Med Res Methodol. (2014) 14:135. doi: 10.1186/1471-2288-14-135, PMID: 25524443 PMC4383202

[17] Stang A . Critical evaluation of the Newcastle-Ottawa scale for the assessment of the quality of nonrandomized studies in meta-analyses. Eur J Epidemiol. (2010) 25:603–5. doi: 10.1007/s10654-010-9491-z, PMID: 20652370

[18] Higgins JP Thompson SG . Quantifying heterogeneity in a meta-analysis. Stat Med. (2002) 21:1539–58. doi: 10.1002/sim.1186, PMID: 12111919

[19] Egger M Davey Smith G Schneider M Minder C . Bias in meta-analysis detected by a simple, graphical test. BMJ. (1997) 315:629–34. doi: 10.1136/bmj.315.7109.629, PMID: 9310563 PMC2127453

[20] Zhang T . A suite of network commands in stata for network meta-analysis. Chin J Evidence-Based Med. (2015) 15:1352–6. doi: 10.7507/1672-2531.20150221

[21] Wankhede D Yuan T Kloor M Halama N Brenner H Hoffmeister M . Clinical significance of combined tumour-infiltrating lymphocytes and microsatellite instability status in colorectal cancer: a systematic review and network meta-analysis. Lancet Gastroenterol Hepatol. (2024) 9:609–19. doi: 10.1016/S2468-1253(24)00091-8, PMID: 38734024

[22] Bryant A Johnson E Grayling M Hiu S Elattar A Gajjar K . Residual disease threshold after primary surgical treatment for advanced epithelial ovarian cancer, part 1: A systematic review and network meta-analysis. Am J Ther. (2023) 30:e36–55. doi: 10.1097/MJT.0000000000001584, PMID: 36608071 PMC9812425

[23] Mende R Vincent FB Kandane-Rathnayake R Koelmeyer R Lin E Chang J . Analysis of serum interleukin (IL)-1β and IL-18 in systemic lupus erythematosus. Front Immunol. (2018) 9:1250. doi: 10.3389/fimmu.2018.01250, PMID: 29930551 PMC5999794

[24] Liu X Bao C Hu D . Elevated interleukin-18 and skewed Th1:Th2 immune response in lupus nephritis. Rheumatol Int. (2012) 32:223–9. doi: 10.1007/s00296-010-1609-9, PMID: 20963419

[25] Tucci M Quatraro C Lombardi L Pellegrino C Dammacco F Silvestris F . Glomerular accumulation of plasmacytoid dendritic cells in active lupus nephritis: role of interleukin-18. Arthritis Rheumatol. (2008) 58:251–62. doi: 10.1002/art.23186, PMID: 18163476

[26] Calvani N Richards HB Tucci M Pannarale G Silvestris F . Up-regulation of IL-18 and predominance of a Th1 immune response is a hallmark of lupus nephritis. Clin Exp Immunol. (2004) 138:171–8. doi: 10.1111/j.1365-2249.2004.02588.x, PMID: 15373921 PMC1809179

[27] Sigdel KR Duan L Wang Y Hu W Wang N Sun Q . Serum cytokines Th1, Th2, and Th17 expression profiling in active lupus nephritis-IV: from a Southern Chinese Han population. Mediators Inflamm. (2016) 2016:4927530. doi: 10.1155/2016/4927530, PMID: 27738386 PMC5055982

[28] Hu D Liu X Chen S Bao C . Expressions of IL-18 and its binding protein in peripheral blood leukocytes and kidney tissues of lupus nephritis patients. Clin Rheumatol. (2010) 29:717–21. doi: 10.1007/s10067-010-1386-6, PMID: 20140691

[29] Shimizu C Fujita T Fuke Y Ito K Satomura A Matsumoto K . High circulating levels of interleukin-18 binding protein indicate the severity of glomerular involvement in systemic lupus erythematosus. Mod Rheumatol. (2012) 22:73–9. doi: 10.3109/s10165-011-0471-2, PMID: 21656327

[30] Wong CK Ho CY Li EK Tam LS Lam CW . Elevated production of interleukin-18 is associated with renal disease in patients with systemic lupus erythematosus. Clin Exp Immunol. (2002) 130:345–51. doi: 10.1046/j.1365-2249.2002.01989.x, PMID: 12390326 PMC1906516

[31] Wu CY Yang HY Yao TC Liu SH Huang JL . Serum IL-18 as biomarker in predicting long-term renal outcome among pediatric-onset systemic lupus erythematosus patients. Med (Baltimore). (2016) 95:e5037. doi: 10.1097/MD.0000000000005037, PMID: 27749566 PMC5059068

[32] Ibrahim NK Allawi AAD Ghudhaib KK Hammoudi FA . Estimation of some immunological markers of Iraqi patients in systemic lupus erythematosus with lupus nephritis. Medico Legal Update. (2020) 20:643–9.

[33] Jafari-Nakhjavani MR Abedi-Azar S Nejati B . Correlation of plasma interleukin-18 concentration and severity of renal involvement and disease activity in systemic lupus erythematosus. J Nephropathol. (2016) 5:28–33. doi: 10.15171/jnp.2016.05, PMID: 27047807 PMC4790184

[34] Bakry E Rahman SA . Interleukin-18 as a biomarker of subclinical lupus nephritis. Arch Rheumatol. (2015) 30:6–15. doi: 10.5606/ArchRheumatol.2015.4675

[35] Koenig KF Groeschl I Pesickova SS Tesar V Eisenberger U Trendelenburg M . Serum cytokine profile in patients with active lupus nephritis. Cytokine. (2012) 60:410–6. doi: 10.1016/j.cyto.2012.07.004, PMID: 22846145

[36] Mohsen MA Karim SAA Abbas TM Maha A . Serum interleukin-18 levels in patients with systemic lupus erythematosus: Relation with disease activity and lupus nephritis. Egyptian Rheumatologist. (2013) 35:45–51. doi: 10.1016/j.ejr.2012.09.005

[37] Chen DY Hsieh CW Chen KS Chen YM Lin FJ Lan JL . Association of interleukin-18 promoter polymorphisms with WHO pathological classes and serum IL-18 levels in Chinese patients with lupus nephritis. Lupus. (2009) 18:29–37. doi: 10.1177/0961203308094559, PMID: 19074166

[38] Farid A Hany A Khaled A Safwat G . Cytokines and autoantibodies profile during systemic lupus erythematosus and psoriasis diseases in Egypt. . J King Saud Univ - Sci. (2022) 34:102007. doi: 10.1016/j.jksus.2022.102007

[39] Kahlenberg JM Kaplan MJ . The inflammasome and lupus: another innate immune mechanism contributing to disease pathogenesis? Curr Opin Rheumatol. (2014) 26:475–81. doi: 10.1097/BOR.0000000000000088, PMID: 24992143 PMC4153426

[40] Wong CK Lit LC Tam LS Li EK Wong PT Lam CW . Hyperproduction of IL-23 and IL-17 in patients with systemic lupus erythematosus: implications for Th17-mediated inflammation in auto-immunity. Clin Immunol. (2008) 127:385–93. doi: 10.1016/j.clim.2008.01.019, PMID: 18373953

[41] Groom JR Fletcher CA Walters SN Grey ST Watt SV Sweet MJ . BAFF and MyD88 signals promote a lupuslike disease independent of T cells. J Exp Med. (2007) 204:1959–71. doi: 10.1084/jem.20062567, PMID: 17664289 PMC2118661

[42] Novick D Elbirt D Miller G Dinarello CA Rubinstein M Sthoeger ZM . High circulating levels of free interleukin-18 in patients with active SLE in the presence of elevated levels of interleukin-18 binding protein. J Autoimmun. (2010) 34:121–6. doi: 10.1016/j.jaut.2009.08.002, PMID: 19699611

[43] Favilli F Anzilotti C Martinelli L Quattroni P De Martino S Pratesi F . IL-18 activity in systemic lupus erythematosus. Ann N Y Acad Sci. (2009), 1173:301–9. doi: 10.1111/j.1749-6632.2009.04742.x, PMID: 19758166

[44] Novick D . IL-18 and IL-18BP: A unique dyad in health and disease. Int J Mol Sci. (2024) 25:13505. doi: 10.3390/ijms252413505, PMID: 39769266 PMC11727785

[45] Novick D Kim S Kaplanski G Dinarello CA . Interleukin-18, more than a Th1 cytokine. Semin Immunol. (2013) 25:439–48. doi: 10.1016/j.smim.2013.10.014, PMID: 24275602

[46] Dinarello CA . Overview of the IL-1 family in innate inflammation and acquired immunity. Immunol Rev. (2018) 281:8–27. doi: 10.1111/imr.12621, PMID: 29247995 PMC5756628

