# Supplementary material for: Interleukin-18 in lupus nephritis: a meta-analysis of cytokine signaling dysregulation in immune-mediated nephropathy
Source: Front Immunol. 2025 Oct 10;16:1631728. doi: 10.3389/fimmu.2025.1631728 (PMC12549602; doi:10.3389/fimmu.2025.1631728)
Supplement: Supplementary file 1 [file DataSheet1.docx]

Supplementary Information

**Interleukin-18 in Lupus Nephritis: a Meta-Analysis of Cytokine Signaling Dysregulation in Immune-Mediated Nephropathy**

**Supplement Table S1. Full Search Strategy**

| **Datebase** | **Query** | **Last search Date** | **Record** |
| --- | --- | --- | --- |
| PubMed | ((((((plasma) OR serum) OR circulate)) AND ((((Interleukin-18 [MeSH Terms]) OR IL-18) OR interleukin-18)))) OR ((((Interleukin-18 binding protein [MeSH Terms]) OR IL-18BP) OR interleukin-18BP))) AND ((((lupus nephritis[MeSH Terms]) OR lupus nephritis) OR LN)) | 07/10/2025 | 71 |
| Embase | ('interleukin 18'/exp OR 'interleukin 18' OR 'interleukin 18 binding protein'/exp OR 'interleukin 18 binding protein') AND 'lupus erythematosus nephritis' | 07/10/2025 | 202 |
| Web of Science | ((IL-18 OR interleukin-18) OR (IL-18BP OR IL-18 binding protein)) AND (LN OR lupus nephritis) | 07/10/2025 | 202 |
| Wiley Online Library | (IL*18 OR interleukin*18 OR IL*18BP OR interleukin*18BP) AND (LN OR lupus nephritis) | 07/10/2025 | 164 |
| MEDLINE | ((((((plasma) OR serum) OR circulate)) AND ((((Interleukin-18 [MeSH Terms]) OR IL-18) OR interleukin-18)))) OR ((((Interleukin-18 binding protein [MeSH Terms]) OR IL-18BP) OR interleukin-18BP))) AND ((((lupus nephritis[MeSH Terms]) OR lupus nephritis) OR LN)) | 07/10/2025 | 341 |
| Scopus | ( IL*18 OR interleukin*18 ) OR ( IL*18BP OR interleukin*18BP ) AND ( LN OR lupus nephritis ) | 07/10/2025 | 134 |
| Cochrane Library | ((IL-18 OR interleukin-18) OR (IL-18BP OR IL-18 binding protein)) AND (LN OR lupus nephritis) | 07/10/2025 | 6 |

A systematic literature search was performed in the these databases.The queries were changed accordingly to adapt to the specific searching requirements of each database.

**Supplement Table S2. Detailed scores of Newcastle–Ottawa-Scale (NOS) criteria**

| **Authors**  **(years)** | **Selection** | | | | **Comparability** | | **Exposure** | | | **Overall**  **quality** |
| --- | --- | --- | --- | --- | --- | --- | --- | --- | --- | --- |
|  | Adequate  definition of the cases | Representativeness of the cases | Seection of controls | Definition of controls | Comparability of cases and controls on the basis of the design or analysis | | Ascertainment of exposure | Same method of ascertainment for cases and controls | Non-  response |  |
| RachelMende  (2008) | 1 | 1 | 1 | 1 | 1 | 1 | 0 | 1 | 1 | 8 |
| Xiaoqian Liu  (2012) | 1 | 1 | 1 | 1 | 1 | 1 | 0 | 1 | 1 | 8 |
| N.CALVAI  (2016) | 1 | 1 | 1 | 1 | 1 | 1 | 0 | 1 | 1 | 8 |
| Keshav Raj Sigdel  (2015) | 1 | 1 | 1 | 1 | 1 | 1 | 0 | 1 | 1 | 8 |
| Chie Shimizu  (2018) | 1 | 1 | 1 | 1 | 1 | 1 | 0 | 1 | 1 | 8 |
| C.K. WONG  (2002) | 1 | 1 | 1 | 1 | 1 | 1 | 0 | 1 | 1 | 8 |
| Chao-Yi Wu  (2016) | 1 | 1 | 1 | 1 | 1 | 1 | 1 | 1 | 1 | 9 |
| Noor Alhuda Kh  (2020) | 1 | 1 | 1 | 1 | 1 | 1 | 0 | 1 | 1 | 8 |
| V Umare  (2019) | 1 | 1 | 1 | 1 | 0 | 0 | 0 | 1 | 1 | 7 |
| Katrin Franzika Koenig  (2012) | 1 | 1 | 1 | 0 | 0 | 1 | 0 | 1 | 1 | 7 |
| Alyaa Farid  (2022) | 1 | 1 | 1 | 1 | 1 | 1 | 1 | 1 | 1 | 9 |

**Supplement Table S3. Detailed scores of Agency for Health-care Research and Quality (AHRQ) criteria.**

| **Authors**  **(years)** | **AHRQ** | | | | | | | | | | | **Overall**  **quality** |
| --- | --- | --- | --- | --- | --- | --- | --- | --- | --- | --- | --- | --- |
|  | 1 | 2 | 3 | 4 | 5 | 6 | 7 | 8 | 9 | 10 | 11 |  |
| MarcoTucci  (2008) | 1 | 1 | 1 | 1 | 1 | 1 | 1 | 0 | 0 | 1 | 0 | 8 |
| Dawei Hu  (2010) | 1 | 1 | 1 | 1 | 1 | 1 | 1 | 0 | 0 | 1 | 0 | 8 |
| Mohammad Reza(2016) | 1 | 1 | 1 | 1 | 1 | 1 | 1 | 0 | 0 | 1 | 0 | 8 |
| SamahAbdel Rahman  (2015) | 1 | 1 | 1 | 1 | 1 | 1 | 1 | 1 | 0 | 1 | 0 | 9 |
| Vinod Umare  (2018) | 1 | 1 | 1 | 1 | 1 | 1 | 1 | 0 | 0 | 1 | 0 | 8 |
| DY Chen  (2009) | 1 | 1 | 1 | 1 | 1 | 1 | 1 | 0 | 0 | 1 | 0 | 8 |
| Mona A. Mohsen  （2013） | 1 | 1 | 1 | 1 | 1 | 1 | 1 | 1 | 0 | 1 | 0 | 9 |
| Dong Liang  (2006) | 1 | 1 | 1 | 1 | 1 | 1 | 0 | 0 | 0 | 0 | 0 | 6 |

**Supplement Table S4. Basic characteristics of included 11 studies in SLE with LN and SLE without LN.**

| Author | Year | Region | Race | Study design | Study quality | Testing method | Sample  type | SLE without LN patirents | | | | SLE with LN patients | | | |
| --- | --- | --- | --- | --- | --- | --- | --- | --- | --- | --- | --- | --- | --- | --- | --- |
|  |  |  |  |  |  |  |  | Size | Mean age(y) | Sex  (F/M) | IL-18  (pg/ml) | Size | Mean age(y) | Sex  (F/M) | IL-18  (pg/ml) |
| Rachel Mende  [24] | 2018 | Australian | Mixed | PC | 8 | ELISA | Serum | 126 | 44.9 | NP | 222.40  (±168.74) | 58 | 44.9 | NP | 312.98  (±174.02) |
| Marco Tucci  [26] | 2008 | Italy | No  Asian | CC | 8 | ELISA | Serum | 17 | NP | NP | 250.00  (±200.00) | 35 | 39.1 | 17/1 | 814.00  (±185.00) |
| N.CALVANI  [27] | 2004 | America  Italy | Mixed | RC | 8 | ELISA | Serum | 72 | 42.1 | 68/4 | 340.00  (±177.60) | 61 | 36.9 | 53/8 | 736.90  (±447.80) |
| C.K. WONG  [31] | 2002 | Hong Kong | Asian | RC | 8 | ELISA | Plasma | 37 | 39.3 | 37/0 | 199.05  (±116.69) | 35 | 39.1 | 34/1 | 249.46  (±192.16) |
| Chao-Yi Wu  [32] | 2016 | Taiwan | Asian | PC | 9 | ELISA | Serum | 31 | 13.10 | 29/2 | 481.92  (±82.18) | 65 | 12.56 | 58/7 | 849.20  (±110.71) |
| N.A.K  Ibrahim  [33] | 2020 | Iraq | No  Asian | RC | 8 | ELISA | Serum | 40 | 29.37 | NP | 378.00  (±38.00) | 40 | 31.05 | 32/8 | 896.00  (±134.00) |
| V  Umare  [34] | 2019 | India | Asian | RC | 7 | ELISA | Serum | 91 | 28 | NP | 468.30  (±242.90) | 109 | 28 | NP | 546.40  (±237.20) |
| 1. R.   J-N  [35] | 2016 | Iran | No  Asian | CC | 8 | ELISA | Serum | 14 | NP | NP | 69.21  (±27.74) | 99 | 30.74 | 92/7 | 649.58  (±663.72) |
| K.F  Koenig  [37] | 2012 | Switzerland | No  Asian | PC | 9 | Cytokine  multiplex  assay | Serum | 14 | 43 | 12/2 | 282.23  (±290.78) | 12 | 31 | 8/4 | 332.83  (±357.66) |
| Mona A. Mohsen  [38] | 2013 | Egypt | No  Asian | CC | 9 | ELISA | Serum | 31 | 36.03 | 28/3 | 56.44  (±23.59) | 41 | 32.41 | 32/9 | 156.13  (±57.86) |
| DY  Chen  [39] | 2009 | Taiwan | Asian | CC | 8 | ELISA | Serum | 64 | 33.1 | 58/8 | 302.07  (±194.41) | 101 | 31.8 | 92/9 | 347.19  (±222.70) |

**Supplement Table S5. Concentration of IL-18 in 6 studies included LN staging.**

| Author | Year | Healthy controls | | LN-II patirents | | LN-III patirents | | LN-IV patirents | | LN-V patirents | |
| --- | --- | --- | --- | --- | --- | --- | --- | --- | --- | --- | --- |
|  |  | Size | IL-18  (pg/ml) | Size | IL-18  (pg/ml) | Size | IL-18  (pg/ml) | Size | IL-18  (pg/ml) | Size | IL-18  (pg/ml) |
| Marco Tucci  [26] | 2008 | 41 | 150.00  (±25.00) | 1 | 337.00  (±0) | 5 | 415.00  (±35.00) | 8 | 871.00  (±75.00) | 4 | 884.00  (±349.00) |
| Chie Shimizu  [30] | 2012 | 32 | 244.00  (±24.00) | 13 | 967.00  (±386.00) | 9 | 1271.00  (±295.00) | 12 | 570.00  (±239.00) | 11 | 315.00  (±56.00) |
| S.A.R. EL. B  [36] | 2015 | 20 | 209.30  (±92.30) | 12 | 476.80  (±85.80) | 16 | 609.20  (±112.90) | 9 | 833.10  (±51.80) | NP | NP |
| Mona A. Mohsen  [38] | 2013 | 15 | 112.01  (±48.23) | 5 | 121.90  (±60.44) | 12 | 152.53  (±46.01) | 17 | 167.01  (±67.41) | 7 | 107.34  (±60.66) |
| DY  Chen  [39] | 2009 | 174 | 77.79  (±39.77) | NP | NP | 26 | 283.79  (±190.99) | 45 | 406.77  (±256.85) | 30 | 331.89  (±242.09) |
| K.R Sigdel  [28] | 2016 | 24 | 11.86  (±3.27) | NP | NP | 12 | 33.21  (±12.22) | 32 | 66.32  (±25.87) | 5 | 29.73  (±8.08) |

**Supplement Figure S6. Egger’s linear regression test in circulating IL-18 levels between LN patients and healthy controls.**

**
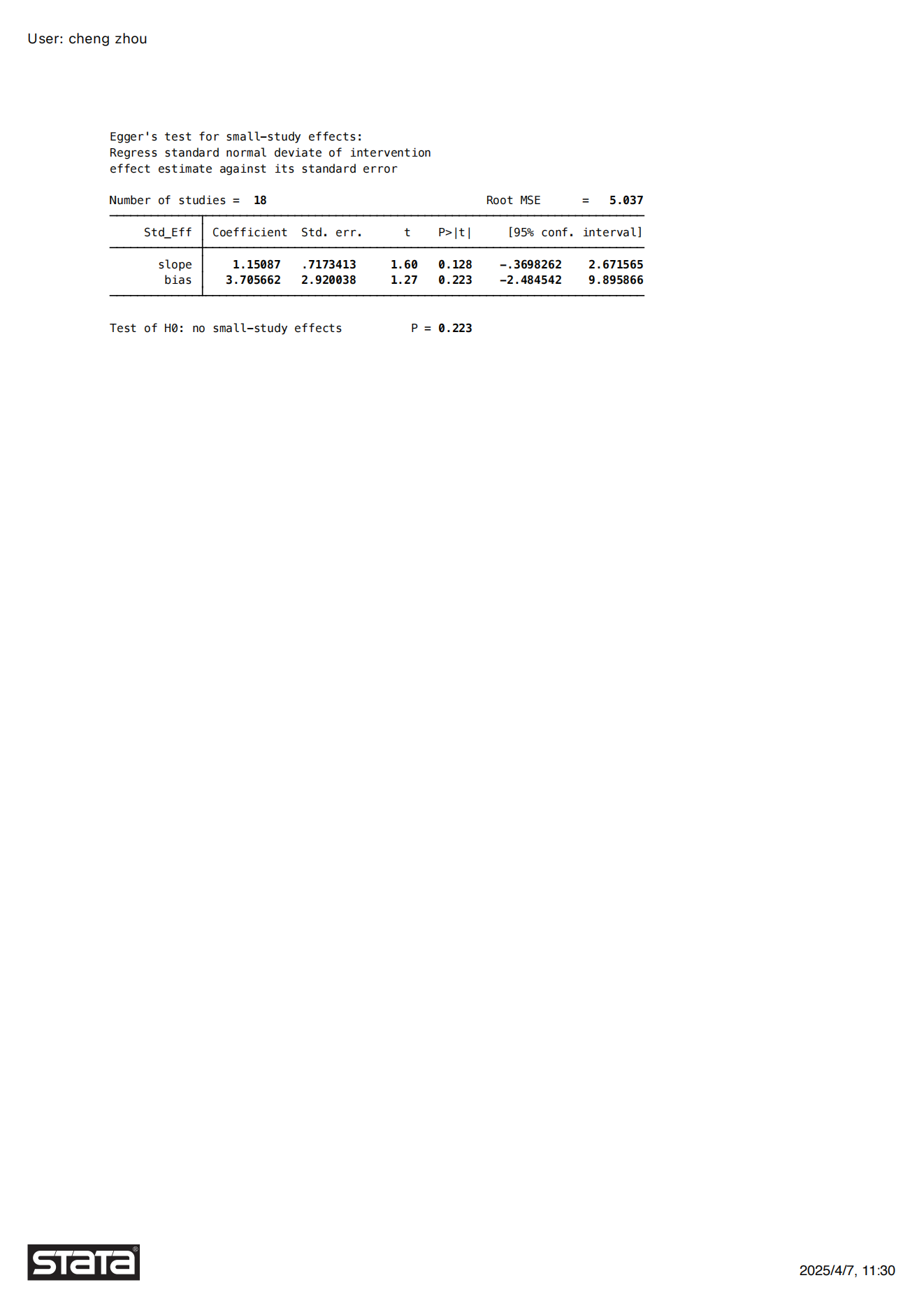
**

**Supplement Figure S7. Meta regression model in different variables, including publication year, mean age of LN groups, disease duration, sample type, study design and ethnicity .**

**
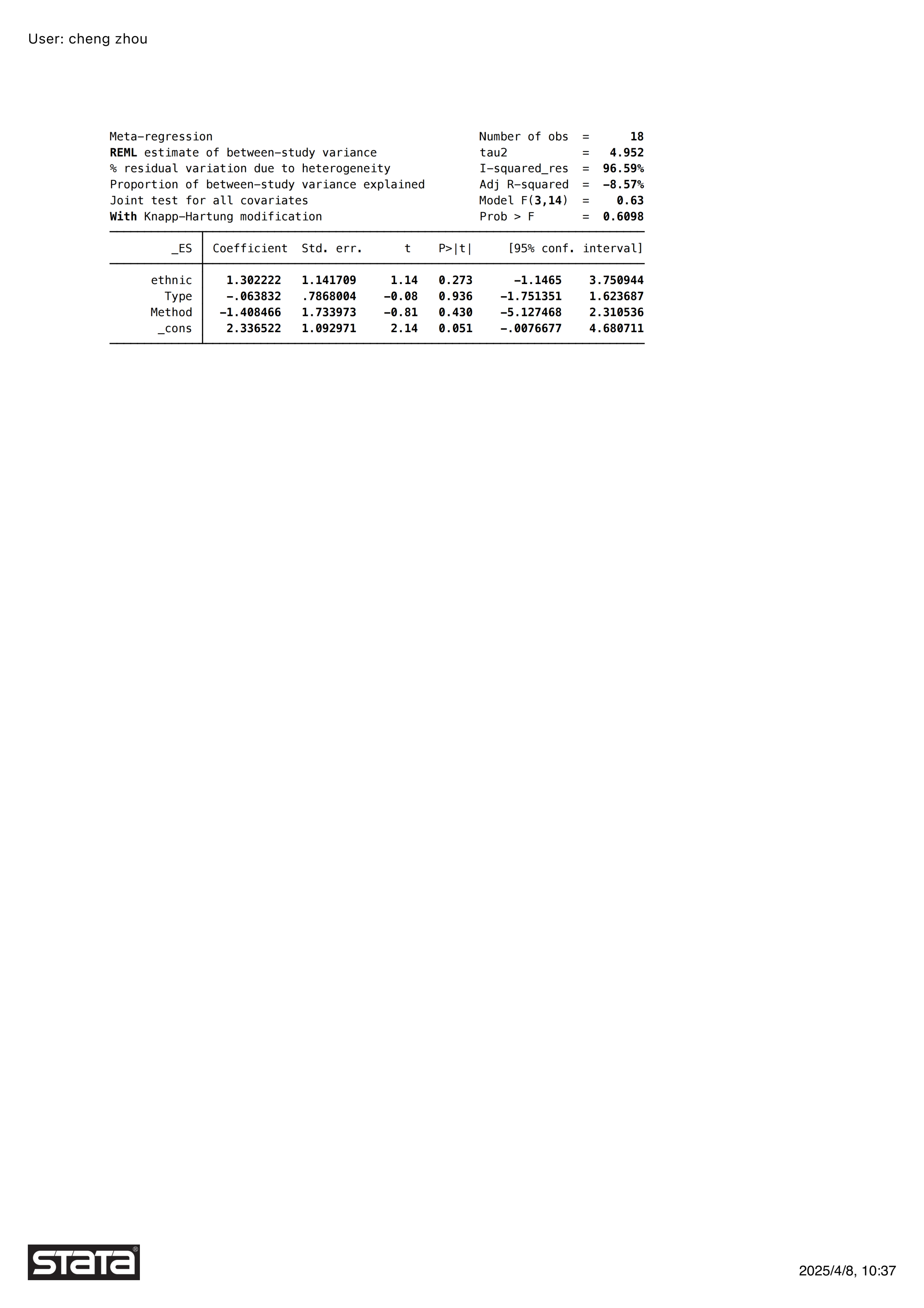
**

**
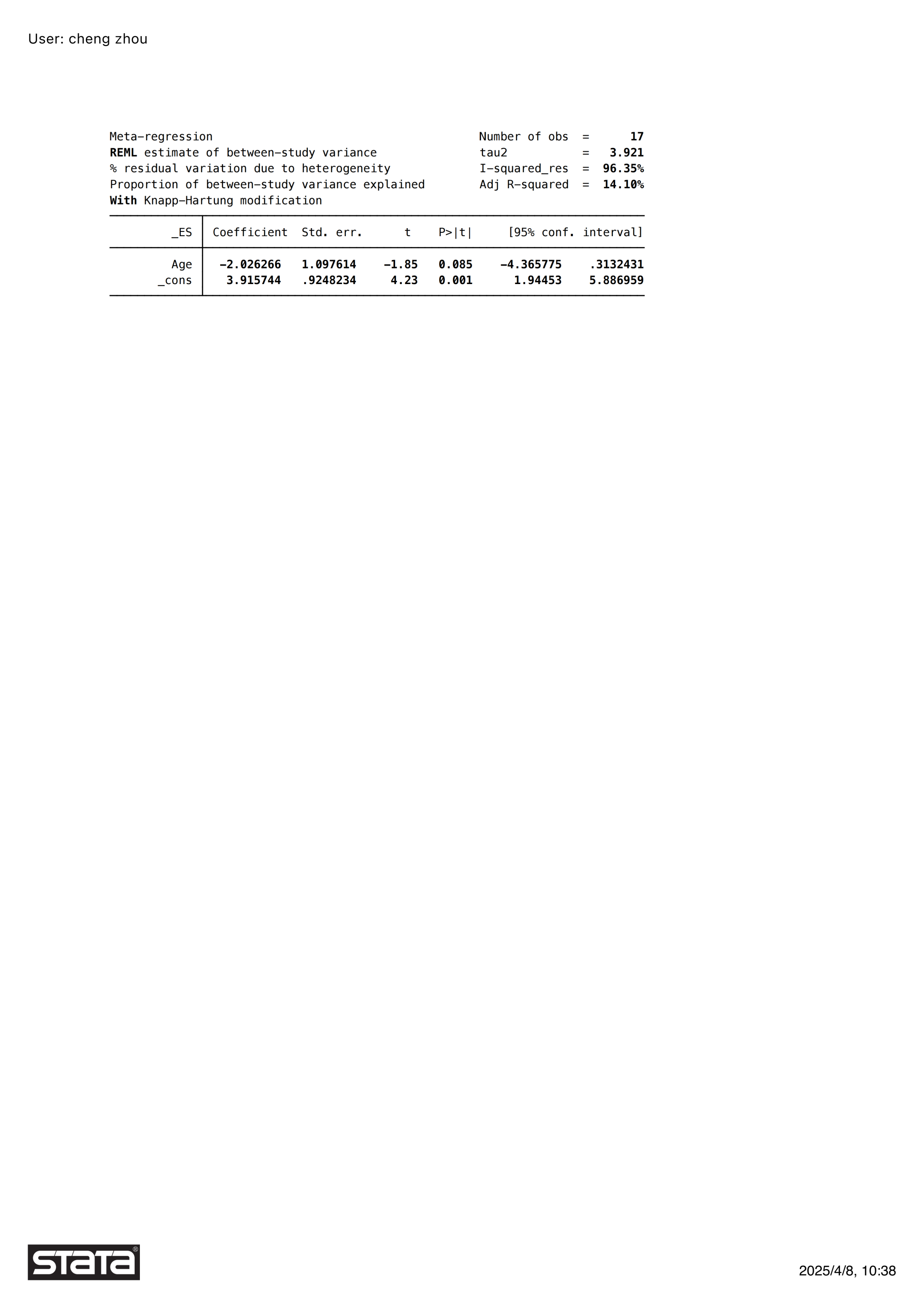
**

**
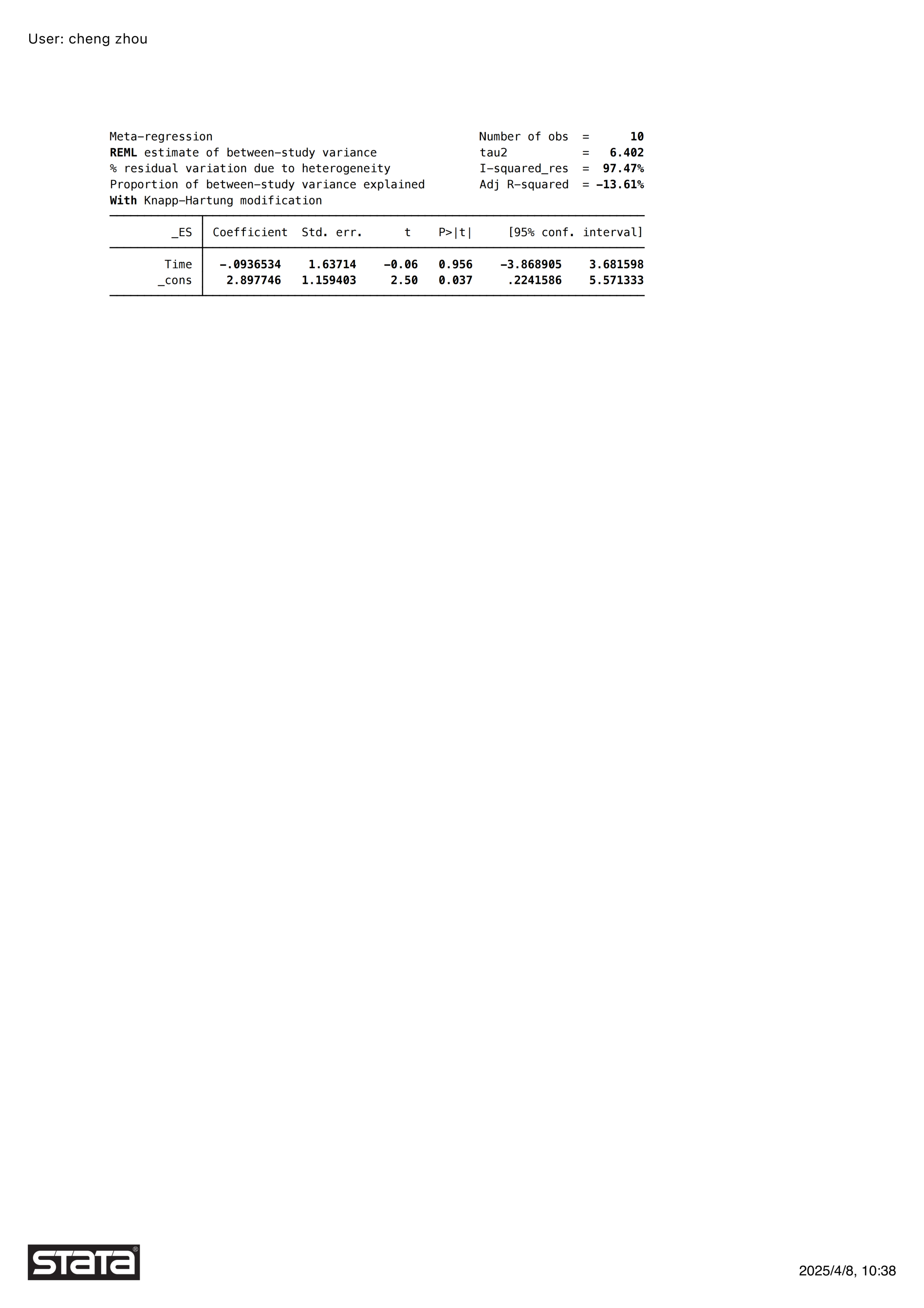
**
